# Evaluating the effect of nutritional support in geriatric inpatients classified by the GLIM criteria

**DOI:** 10.1016/j.jnha.2025.100585

**Published:** 2025-05-23

**Authors:** Shan Jiang, Xiling Chen, Lan Ma, Qihao Guo, Lan Luo, Yuehui Wang, Xuan Qu, Jiaojiao Li, Liping An, Wei Huang, Yonghua Wu, Hongyu Zhang, Cuntai Zhang, Yun Fan, Songbai Zheng, Jian Cao, Xiaohong Liu

**Affiliations:** a Department of Geriatrics, Chinese Academy of Medical Sciences, Peking Union Medical College Hospital, No. 1 Shuaifuyuan, Dongcheng District, Beijing 100730, China; b Department of Geriatrics, The Second Affiliated Hospital of Zhengzhou University, No.2, Jingba Road, Jinshui District, Zhengzhou 450003, Henan, China; c Department of Geriatrics, The Second Affiliated Hospital of Harbin Medical University, No.246, Xuefu Road, Nangang District, Harbin 150086, Heilongjiang, China; d Department of Geriatrics, Shanghai Sixth People's Hospital, Shanghai Jiao Tong University School of Medicine, No.600, Yishan Road, Xuhui District, Shanghai 200233, China; e Department of Geriatrics, Affiliated Hospital of Nantong University, No. 20, Xisi Road, Chongshan District, Nantong 226006, Jiangsu, China; f Department of Geriatrics, The First Hospital of Jilin University, No. 1, Xinmin Road, Chaoyang District, Changchun 130021, Jilin, China; g Department of Geriatrics, Heilongjiang Provincial Hospital, No. 82, Zhongshan Road, Xiangfang District, Harbin 151100, Heilongjiang, China; h Department of Geriatrics, Tangshan Gongren Hospital, No. 27, Wenhua Road, Lubei District, Tangshan 063099, Hebei, China; i Department of Geriatrics, Suzhou Municipal Hospital, No. 26, Daoqian Road, Gusu District, Suzhou 215002, Jiangsu, China; j Department of Geriatrics, Qilu Hospital of Shandong University, No. 107, Wenhua West Road, Lixia District, Jinan 250012, Shandong, China; k Department of Geriatrics, Huazhong University of Science and Technology Tongji Hospital, No. 1095, Jiefang Avenue, Qiaokou District, Wuhan 430030, Hubei, China; l Department of Geriatrics, Beijing Hospital, No. 1, Dahua Road, Dongcheng District, Beijing 100005, China; m Department of Geriatrics, Huadong Hospital Affiliated to Fudan University, No. 221, Yan'an West Road, Jingan District, Shanghai 200040, China; n Department of Geriatrics, The Second Medical Center, The National Clinical Research Center for Geriatric Diseases, Chinese People’s Liberation Army General Hospital, No. 28, Fuxing Road, Haidian District, Beijing 100853, China

**Keywords:** GLIM criteria, Malnutrition, Nutritional support therapy, Older inpatients

## Abstract

**Objectives:**

In this study, we aimed to explore the impact of nutrition therapy on clinical outcomes for patients classified according to the Global Leadership Initiative on Malnutrition (GLIM) criteria.

**Design:**

Prospective, multicenter cohort study.

**Setting:**

This study was conducted from September 2020 to December 2022 across 28 geriatric centers in China.

**Participants:**

A total of 862 patients aged ≥65 years were included.

**Intervention:**

All participating physicians completed a 6-h training on nutritional support, following international guidelines before the study. Patients had a nutritional risk screening 2002 score ≥3 points within 48 h of admission. Physicians determined specific nutritional support regimens.

**Measurements:**

GLIM assessments were conducted after enrollment. Nutritional and functional statuses were evaluated at baseline and 90 days after admission. Clinical outcomes—mortality, readmission, new infections, and falls—were documented after 90 days.

**Results:**

Compared to 108 patients without malnutrition per the GLIM criteria, 754 malnourished patients showed lower weight, body mass index (BMI), and Mini Nutritional Assessment-Short Form (MNA-SF) scores and significant reductions in grip strength, calf circumference, and Barthel activities of daily living (ADLs) index. The percentage of patients with adequate caloric intake at the 90-day follow-up was 70.7% (*n* = 533) and 67.6% (*n* = 73) in the malnutrition and non-malnutrition groups (*p* = 0.51) and that of patients with adequate protein intake was 65.9% (*n* = 497) and 58.3% (*n* = 63), respectively (*p* = 0.12). Moreover, malnourished patients showed significant improvements in body weight, BMI, MNA-SF scores, calf circumference, and Barthel ADL index compared to those without malnutrition. Malnourished patients also had lower risks of readmission and falls at follow-up.

**Conclusion:**

Among older inpatients at nutritional risk, those with malnutrition classified according to the GLIM criteria benefited from nutritional support, demonstrating improved BMI, MNA-SF scores, calf circumference, and Barthel ADL index, as well as reduced readmission rates and incidence of falls.

## 1 Introduction

Malnutrition is a common condition in geriatric patients, with a particularly high incidence among hospitalized older patients [1,2]. In the evaluation of patients aged ≥65 years from 30 major hospitals in 14 cities in China, the Chinese Society for Parenteral and Enteral Nutrition (CSPEN) reported that approximately 50% of inpatients were at nutritional risk (Nutritional Risk Screening 2002 [NRS2002] score ≥3) [3]. Malnutrition results in adverse clinical outcomes, including increased complications, prolonged hospital stays, readmissions, disabilities, decreased quality of life, mortality among older patients, and increased burdens on healthcare systems, including increased healthcare costs [[4], [5], [6], [7]]. However, malnutrition diagnosis remains a significant challenge in healthcare settings, particularly among non-nutritional experts. Varying diagnostic criteria and emphasis levels have resulted in inconsistent nutritional support therapies (NSTs) and difficulty evaluating nutritional efficacy. The NRS2002 system was initially released in 2002 by the European Society for Clinical Nutrition and Metabolism (ESPEN) [8,9] and introduced in China in 2005 [10]. The NRS2002 system was recommended by the CSPEN as the first option for nutritional risk screening [11], which then influenced China’s social medical insurance system’s coverage of the diagnosis and treatment of malnutrition.

In 2019, the Global Leadership Initiative on Malnutrition (GLIM) proposed new universal criteria to standardize malnutrition diagnosis in adults based on a two-step model of risk screening and diagnostic assessment [12]. The consensus recommended that countries conduct prospective validation studies. Since the GLIM criteria consensus was proposed, studies have mainly compared the effectiveness of these criteria with that of other malnutrition assessment tools and their predictive validity on clinical outcomes in various populations [13,14]. However, few observational trials have specifically investigated the efficacy and impact of NSTs on clinical outcomes in older inpatients classified according to the GLIM criteria. This is important because this large and growing segment of older inpatients is at nutritional risk. Therefore, identifying appropriate assessment modalities for this population is crucial to facilitate effective NSTs and potentially prevent adverse clinical outcomes and nutritional and functional decline.

In this multicenter prospective cohort study of inpatients in geriatric wards in China, we aimed to investigate the effects of NSTs on clinical outcomes in patients classified according to the GLIM criteria to explore the predictive value of this new approach for diagnosing malnutrition.

## 2 Methods

### 2.1 Patient selection

This prospective multicenter cohort study was designed by the Department of Geriatrics at Peking Union Medical College Hospital (PUMCH), and it consisted of patients from the geriatric wards of 28 tertiary hospitals in various provinces and cities in China. All centers adopted a validated screening tool for malnutrition based on the NRS 2002 score [9]. Nutritional risk screening included assessment of the patient’s nutritional status (based on weight loss, body mass index [BMI], and general condition or food intake) and disease severity (stress metabolism). Each risk predictor is scored from 0 to 3 points, and patients receive an extra point if they are >70 years of age. Eligible inpatients were consecutively enrolled between September 2020 and December 2022. The enrollment criteria included age ≥65 years, nutritional risk (NRS2002 ≥ 3 points), and a hospital stay of at least 2 days. The exclusion criteria included terminal disease (estimated survival time ≤3 months), transfer to another department during hospitalization, contraindications (such as hemodynamic instability), and previous participation in another nutritional research project within 6 months.

This study was conducted in accordance with the principles embodied in the Declaration of Helsinki. The study design was approved by the Research Ethics Committee of PUMCH (ZS-2429; date of ethics approval: August 20, 2020) and registered at ClinicalTrials.gov (NCT04751032). Written informed consent was obtained from all patients.

### 2.2 Procedures

The principal investigator trained the participating geriatricians on the study protocol, implementation steps, and timelines. All participating physicians attended a 6-h training session regarding nutritional support during hospital stay and after discharge, as stipulated in international guidelines [15,16] prior to the study. First, the NRS2002 system was used to enroll inpatients; next, the GLIM criteria were used to diagnose malnutrition and determine its severity. Patients were diagnosed with malnutrition if any of the three phenotypic criteria (unintentional weight loss, low BMI, and reduced muscle mass) and either of the two etiological criteria (reduced food intake or assimilation and disease burden/inflammation) were met (Table 1). According to clinical guidelines, patients at nutritional risk or diagnosed with malnutrition should receive NSTs. The nutritional regimen recommended setting daily caloric requirement and protein intake at 25–30 kcal/kg and 1.0–1.2 g/kg, respectively, with a minimum nutritional support target of ≥75%. Each participating center’s geriatric team included a trained nutritionist who assessed participants’ dietary energy and protein intake to assist the geriatrician in developing individualized nutritional support regimens. The investigator first adjusted the diet to fortified foods based on the patient's preferences and added oral nutritional supplements (ONS) between meals. When dietary intake and ONS could only provide <50% of the target requirement within 5 days, additional nutritional support through enteral tube feeding or parenteral feeding was recommended. During hospitalization, a trained nutritionist re-assessed nutritional intake weekly based on each patient's dietary records. Upon discharge, geriatricians developed an individualized outpatient follow-up and out-of-hospital nutrition plan. Follow-up was conducted every 2–4 weeks via outpatient visits or telephone consultations to provide dietary counseling and prescribe ONS when necessary. A face-to-face outpatient follow-up was scheduled 90 days post-admission to evaluate each patient’s nutritional intake. Patients were considered to have good compliance if they continued taking ONS as prescribed at the 90-day follow-up.

**Table 1:** GLIM criteria and thresholds for diagnosing malnutrition.

Phenotypic criteria			Etiologic criteria	
Unintentional weight loss (%)	Low BMI (kg/m^2^)	Reduced muscle mass^a^	Reduced food intake or assimilation	Inflammation
>5% within the past 6 months or >10% beyond 6 months	<18.5 if <70 years or <20 if >70 years	Calf circumference (men: <34 cm, women: <33 cm)	<50% of energy requirements >1 week, or any reduction for >2 weeks, or GI symptoms or chronic GI conditions that adversely impacts food intake/absorption/assimilation	Acute disease/injury or chronic disease-related or hsCRP >3 mg/L

Abbreviations: BMI, body mass index; GI, gastrointestinal; GLIM, Global Leadership Initiative on Malnutrition; hsCRP, high-sensitivity C-reactive protein.

a Cut-off points for calf circumference adopted from the literature [27].

### 2.3 Data collection

We constructed a database for the Chinese Nutritional Risk Screening and Intervention Registration Study, which included participant demographics (sex, age, height, weight, and BMI), medical information (acute disease and underlying comorbidities), geriatric assessments (food intake, the Barthel Activities of Daily Living [ADLs] index, handgrip strength, calf circumference, and Mini Nutritional Assessment-Short Form [MNA-SF] score), treatment courses (nutritional treatment pathway, dietary intake, oral nutritional supplementation, laboratory tests including prealbumin, albumin, and high-sensitivity C-reactive protein [hsCRP] levels), and follow-up data reported during the study. Nutritional status was evaluated using weight, BMI, and MNA-SF [17]. Muscle strength was systematically assessed by measuring handgrip strength using a dynamometer (Camry EH101 electronic hand dynamometer; Camry, Guangdong, China). The patients were instructed to squeeze the device with maximal exertion for 3 s. The measurements were performed twice for each hand, with at least 30-s rest intervals. The highest muscle strength demonstrated in both hands was used in the analyses. Muscle mass was assessed using calf circumference. Calf circumference was measured using a non-elastic tape while the participant remained in a sitting position with the knee and ankle at right angles. The maximum circumference of both calves was measured perpendicular to the long axis of the calf, and average values were adopted for further analyses. The Barthel ADL index [18] was used to assess functional status. The scores ranged from 0 to 100 points, with higher scores indicating better functional status. The above nutritional- and function-related data were collected within 48 h after admission and at the 90-day follow-up.

The clinical outcome was the occurrence of adverse events within 90 days of admission, including all-cause mortality, readmission, new infections, and falls. New infections were defined as any infection diagnosed by a formal medical institution after discharge, requiring a doctor's prescription of antibiotics as indicated in medical records or diagnosis reports. These infections included those affecting the respiratory, urinary, and gastrointestinal tracts, with supporting evidence, such as increased white blood cell count or elevated hsCRP levels. Falls were recorded based on participant reports, including details on the cause and whether the fall resulted in a fracture.

### 2.4 Statistical analysis

Descriptive analyses at baseline are presented as means ± standard deviations for continuous variables and n (%) for categorical variables. Comparisons between the groups were performed using Student's *t*-test for continuous variables and chi-square test for categorical variables. Pairwise *t*-tests were conducted to evaluate changes in nutrition and function parameters between baseline and 90-day follow-up results, with results reported as estimated changes and 95% confidence intervals (CIs). The endpoint events in each study group are presented as the number of occurrences and proportions. Between-group comparisons were performed using logistic regression, with the non-malnutrition group serving as the reference, adjusted for age, sex, BMI, and NRS2002 score. The odds ratios (ORs), 95% CIs, and p-values associated with the malnutrition group were reported. Missing data were addressed using multiple imputation, followed by sensitivity analyses. All statistical analyses were performed using IBM SPSS Statistics for Windows (version 24.0; IBM Corp, NY, USA). Statistical significance was set at two-tailed p-value ≤0.05.

## 3 Results

**Fig. 1:**
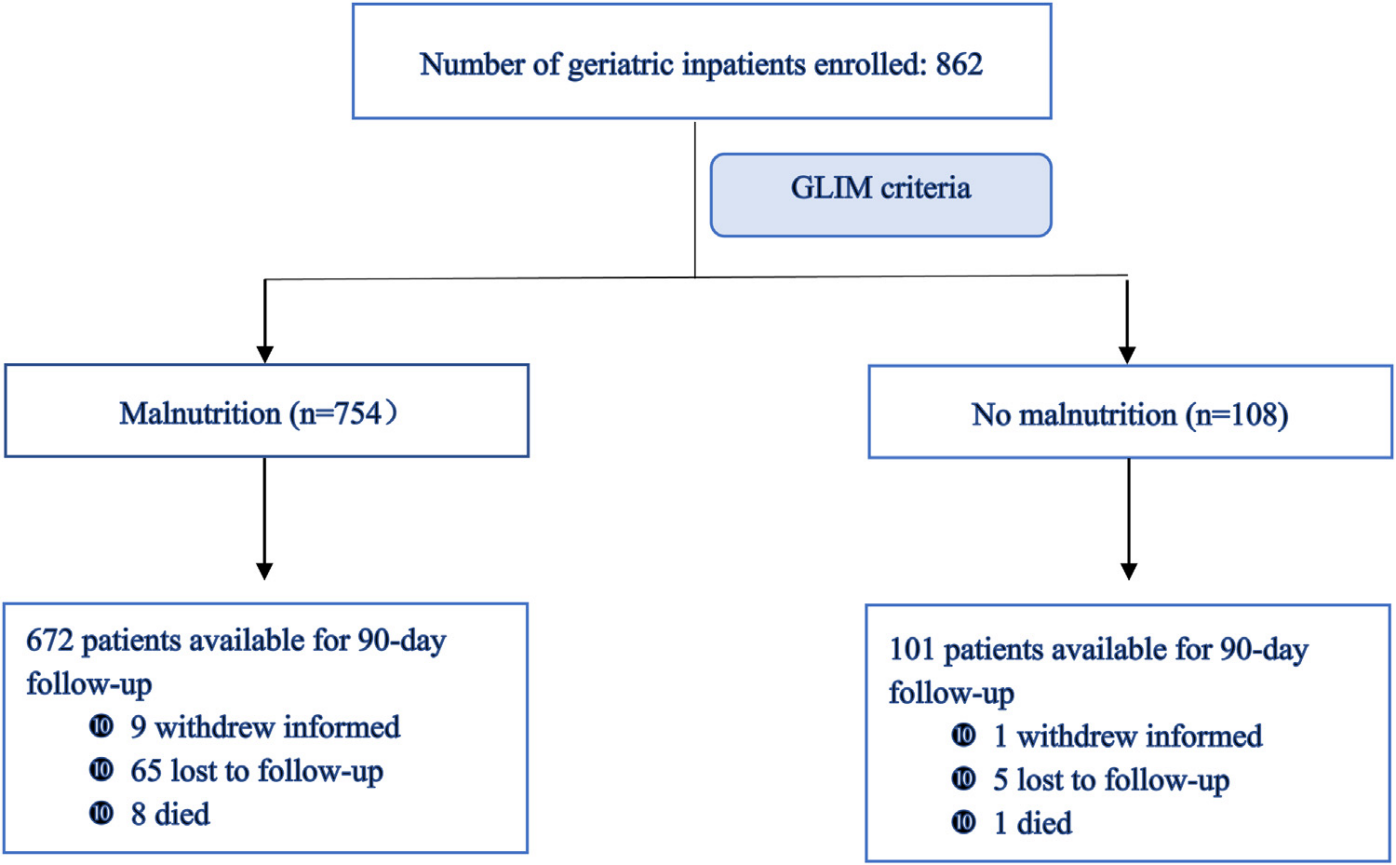
Flowchart of patients throughout the trial. NRS, nutritional risk screening; GLIM, Global Leadership Initiative on Malnutrition.

Initially, 862 inpatients from the geriatric wards of 28 tertiary hospitals met the inclusion criteria (NRS2002 ≥ 3 points). The mean age was 83.5 ± 8.1 years, with 65.2% being male. The most frequent diagnoses necessitating hospital admission included infection, stroke, coronary heart disease, malignant tumors, and heart failure. The patients presented a high burden of comorbidities, such as diabetes, hypertension, cardiovascular, and cerebrovascular disease. A total of 754 (87.5%) patients were diagnosed with malnutrition and 108 (12.5%) without malnutrition, based on the GLIM criteria (Fig. 1). No significant differences were observed in age and sex between the two groups. Regarding the primary admission diagnosis and comorbidities, significant differences were observed only in the prevalence of infection and stroke between the groups. Malnutrition patients exhibited lower weight, BMI, MNA-SF scores, and a significant decline in grip strength, calf circumference, and Barthel ADL index score compared with that exhibited by non-malnutrition patients (*p* < 0.001) (Table 2).

**Table 2:** Baseline demographic and clinical characteristics of patients.

Variable	Entire cohort	Malnutrition	No malnutrition	p*
	*n* = 862	*n* = 754	*n* = 108	
Demographics				
Age (years), mean (SD)	83.5 ± 8.1	83.7 ± 8.1	82.1 ± 7.9	0.05
≥75, *n* (%)	737 (85.5)	651 (86.3)	86 (79.6)	0.06
Male sex, *n* (%)	562 (65.2)	491 (65.1)	71 (65.7)	0.89
Causes of admission, *n* (%)				
Infection	220 (25.5)	184 (24.4)	36 (33.3)	0.04
Stroke	123 (14.3)	103 (13.7)	20 (18.5)	0.17
Coronary heart disease	119 (13.8)	100 (13.3)	19 (17.6)	0.22
Malignant tumor	93 (10.8)	78 (10.3)	15 (13.9)	0.26
Heart failure	37 (4.3)	31 (4.1)	6 (5.6)	0.49
Comorbidities, *n* (%)				
Diabetes	319 (37.0)	277 (36.7)	42 (38.9)	0.66
Hypertension	282 (32.7)	245 (32.5)	37 (34.2)	0.71
Stroke	241 (27.9)	199 (26.4)	42 (38.9)	0.01
Coronary heart disease	166 (19.2)	139 (18.4)	27 (25.0)	0.10
NRS2002 score, *n* (%)				
3	401 (46.5)	338 (44.8)	63 (58.3)	0.01
4	216 (25.1)	189 (25.1)	27 (25.0)	0.98
5	200 (23.2)	182 (24.1)	18 (16.7)	0.08
≥6	45 (5.2)	45 (5.9)	0	0.01
Nutritional assessment				
Caloric intake (kcal), mean (SD)	1349 (611)	1297 (494)	1430 (401)	<0.001
Protein intake (g), mean (SD)	54 (47)	50 (49)	59 (14)	<0.001
Body weight (kg), mean (SD)	57.0 (10.9)	56.8 (10.9)	65.1 (10.1)	<0.001
BMI (kg/m^2^), mean (SD)	20.8 (3.4)	20.8 (3.4)	24.2 (3.3)	<0.001
MNA-SF points, mean (SD)	8.5 (2.8)	8.1 (2.6)	11.3 (2.2)	<0.001
Grip strength (kg), mean (SD)	20.8 (9.1)	19.8 (9.0)	21.3 (8.9)	<0.001
Calf circumference (cm), mean (SD)	29.1 (4.3)	28.5 (4.0)	32.9 (3.8)	<0.001
Barthel ADL score, mean (SD)	68.4 (26.0)	66.9 (26.2)	80.4 (22.0)	<0.001

Abbreviations: ADL, activity of daily living; BMI, body mass index; IQR, interquartile range; MNA-SF, Mini Nutritional Assessment-Short Form; NRS, nutritional risk screening; SD, standard deviation.

* Comparison of baseline parameters in patients with and without malnutrition.

Among the 754 patients diagnosed with malnutrition following the GLIM criteria, nine withdrew informed consent, 65 were lost to follow-up, and eight died by the 90-day follow-up; consequently, 672 participants completed follow-up. Among 108 patients without malnutrition, one withdrew informed consent, five were lost to follow-up, and one died; thus, the final cohort comprised 101 patients. The researchers received training and were well-educated; nevertheless, variations in protocol adherence and patient compliance were observed. At the 90-day follow-up, the percentage of patients with adequate caloric intake was 70.7% (*n* = 533) in the malnutrition group and 67.6% (*n* = 73) in the non-malnutrition group (*p* = 0.51), respectively, and the percentage of patients with adequate protein intake was 65.9% (n = 497) and 58.3% (*n* = 63) (*p* = 0.12), respectively. In the malnutrition group, 482 (63.9%) patients received ONS, 23 (3.1%) received tube feeding, and 23 (3.1%) received parenteral nutrition during hospitalization. Moreover, 315 patients (77%) demonstrated good compliance with ONS at the 90-day follow-up. In the non-malnutrition group, 48 (44.4%) patients received NSTs, primarily ONS, and 42 (38.9%) demonstrated good compliance with ONS at the 90-day follow-up.

At admission, patients with malnutrition exhibited significantly lower mean daily caloric intake (1297 ± 494 kcal vs. 1430 ± 401 kcal) and mean daily protein intake (50 ± 49 g vs. 59 ± 14 g) than those without malnutrition. At the 90-day follow-up, no significant differences were found in daily caloric and protein intake between the groups with and without malnutrition (1480 ± 552 kcal vs. 1453 ± 411 kcal, *p* = 0.78 and 67 ± 35 g vs. 66 ± 26 g, *p* = 0.74, respectively). Furthermore, from baseline to the 90-day follow-up, the increase in mean daily caloric intake was 183 (95% CI: 130–241) kcal in the malnutrition group and 24 (95% CI: −2–101) kcal in the non-malnutrition group (*p* < 0.001). The increase in the mean daily protein intake was 17 g (95% CI: 9–25) and 7 g (95% CI: 1–13) in the malnutrition and non-malnutrition groups, respectively (*p* < 0.001).

The changes in nutritional and functional parameters between the 90-day follow-up and baseline in patients are detailed in Table 3. At the 90-day follow-up, compared with admission values, body weight (difference: 1.4 kg [95% CI: 0.9–1.5]), BMI (difference: 0.5 kg/m^2^ [95% CI: 0.3–0.6]), MNA-SF scores (difference: 1.8 [95% CI: 1.5–1.9]), calf circumference (difference: 0.8 cm [95% CI: 0.6–1.0]), and Barthel ADL index (difference: 3.1 [95% CI: 1.1–4.2]) showed significantly greater improvements in the malnutrition group than in the non-malnutrition group. Notably, by the end of the follow-up period, 211 of the 754 malnourished patients (28.0%) had recovered from malnutrition, whereas 18 of the 108 non-malnourished patients (16.7%) had developed malnutrition.

**Table 3:** Differences in the changes in nutritional and functional parameters between 90-day follow-up and baseline in patients.

Variable	Malnutrition	No malnutrition	p*
	*n* = 754	*n* = 108	
Caloric intake (kcal)			
90-day follow-up, mean (SD)	1480 (552)	1453 (411)	0.78
Increase from baseline (95% CI)	183 (130–241)	24 (−2–101)	<0.001
Protein intake (g)			
90-day follow-up, mean (SD)	67 (35)	66 (26)	0.74
Increase from baseline (95% CI)	17 (9–25)	7 (1–13)	<0.001
Body weight (kg)			
90-day follow-up, mean (SD)	58.2 (10.3)	65.1 (10.5)	<0.001
Increase from baseline (95% CI)	1.4 (0.9–1.5)	−0.04 (−0.8–0.7)	<0.001
BMI (kg/m^2^)			
90-day follow-up, mean (SD)	21.3 (3.4)	24.1 (3.3)	<0.001
Increase from baseline (95% CI)	0.5 (0.3–0.6)	−0.01 (−0.3–0.3)	0.01
MNA-SF points			
90-day follow-up, mean (SD)	9.9 (2.8)	11.8 (2.5)	<0.001
Increase from baseline (95% CI)	1.8 (1.5–1.9)	0.4 (−0.03–0.7)	<0.001
Grip strength (kg)			
90-day follow-up, mean (SD)	21.3 (7.8)	22.2 (7.9)	0.08
Increase from baseline (95% CI)	1.5 (1.2–1.8)	0.9 (0.1–1.7)	0.23
Calf circumference (cm)			
90-day follow-up, mean (SD)	29.3 (4.0)	33.1 (3.9)	<0.001
Increase from baseline (95% CI)	0.8 (0.6–1.0)	0.1 (−0.4–0.2)	<0.001
Barthel ADL score			
90-day follow-up, mean (SD)	70.0 (27.0)	81.5 (21.3)	<0.001
Increase from baseline (95% CI)	3.1(1.1–4.2)	1.1 (−2.5–3.9)	0.01

Abbreviations: ADL, activity of daily living; BMI, body mass index; IQR, interquartile range; MNA-SF, Mini Nutritional Assessment-Short Form; NRS, nutritional risk screening; SD, standard deviation.

* Comparison of 90-day follow-up parameters and the difference between baseline and 90-day follow-up in malnourished and non-malnourished patients.

Regarding clinical outcomes, mortality rates in the malnutrition and non-malnutrition groups were 1.1% (*n* = 8) and 0.9% (*n* = 1), respectively; readmission rates were 12.2% (*n* = 92) and 19.4% (*n* = 21), respectively; rates of new infections were 7.8% (*n* = 59) and 7.4% (*n* = 8), respectively; and fall rates were 2.0% (*n* = 15) and 5.6% (*n* = 6), respectively. The malnutrition group exhibited lower risks of readmission (adjusted OR: 0.42 [95% CI: 0.23–0.75]) and falls (adjusted OR: 0.31 [95% CI: 0.11–0.91]) at the 90-day follow-up compared with that exhibited by the non-malnutrition group, adjusted for age, sex, BMI, and NRS2002 score at baseline. The incidences of mortality and new infections did not show significant differences between the groups (Table 4).

**Table 4:** Effects of NSTs on endpoints in patients with and without malnutrition.

Nutritional status	All-cause mortality		Readmission		New infection		Fall	
	Adjusted OR (95% CI)	*p*	Adjusted OR (95% CI)	*p*	Adjusted OR (95% CI)	*p*	Adjusted OR (95% CI)	*p*
Malnutrition	1.58 (0.44–12.21)	0.52	**0.42 (0.23–0.75)**	**0.01**	0.95 (0.42–2.16)	.92	**0.31 (0.11–0.91)**	**0.03**
No malnutrition	reference		reference		reference		reference	

Abbreviations: CI, confidence interval; OR, odds ratio.

Odds ratios were calculated using logistic regression for binary data, and models were adjusted for age, sex, body mass index, and NRS2002 score.

Bold text indicates values that are statistically significant (p < 0.05).

## 4 Discussion

The participants were recruited from geriatric wards across several provinces and cities in China and were identified as being at nutritional risk, with NRS2002 scores of ≥3. The inpatients were classified following the GLIM criteria. Compared to patients without malnutrition, NST enhanced the nutritional status and functional condition in patients with malnutrition and lowered the incidence of adverse outcomes, such as readmission and falls. Thus, the GLIM criteria might serve as an accurate method for predicting malnutrition diagnosis.

NRS2002 is an evidence-based nutritional risk score used to screen patients for nutritional risks, evaluate the effect of nutritional support, and predict clinical outcomes in hospitalized patients [19]. In China, the medical insurance system only covers inpatients assessed for nutritional risk using NRS2002, making it the preferred screening tool in clinical practice and a basis for NST decision-making. The GLIM framework employs a two-step “screening-diagnosis” approach. After identifying individuals at nutritional risk, it applies the GLIM criteria, focusing on phenotypic and etiologic factors to diagnose malnutrition by identifying individuals with reduced nutrient intake, impaired absorption, or increased nutrient requirements. These criteria provide a more comprehensive approach with high specificity and sensitivity [20]. Since the launch of the GLIM criteria, approximately 400 scientific papers have been published to test their validity and clinical feasibility [14]. However, few prospective studies have specifically examined the efficacy of NSTs on clinical outcomes in older inpatients categorized according to the GLIM criteria. It remains important to explore whether new diagnostic tools can distinguish patients who may benefit from NST and achieve better outcomes.

Notably, the mean age of participants recruited from geriatric wards was over 80 years, with a large proportion exceeding 75 years. Given that older inpatients often have multiple comorbidities, acute inflammatory states, and functional decline, the proportion of GLIM-positive patients among those at nutritional risk was relatively high. Two narrative reviews indicated that applying the GLIM framework demonstrated good criterion and predictive validity in older populations across various clinical settings [21,22]. However, the response to NSTs in older patients diagnosed with malnutrition according to the GLIM criteria remains unclear. Although previous studies have shown that inpatients at nutritional risk according to NRS2002 benefit from NST in terms of improved clinical outcomes [23], these studies did not further categorize patients using the GLIM criteria.

In this present prospective observational study, NSTs were provided to patients at nutritional risk based on nutritional guidelines. We found that malnourished patients compared with non-malnourished patients showed significantly greater improvements in BMI, MNA-SF score, calf circumference, and Barthel ADL index score at the 90-day follow-up after recerving NSTs. In contrast, those at nutritional risk who did not meet the GLIM criteria had a lower disease burden, better baseline nutritional status, greater muscle strength, and higher Barthel ADL index scores. They also demonstrated significantly higher baseline caloric and protein intake than that demonstrated by patients in the malnutrition group. Chavarro-Carvajal et al. indicated that patients who were most likely to experience improvements in nutritional status were those with worse baseline nutritional status, smaller calf circumference, and greater number of comorbidities, suggesting that more favorable changes were observed in those with greater initial impairment [24]. While differences in intervention adherence may have influenced the outcomes, they also reflect the real-world application of the GLIM criteria in clinical decision-making. In practice, NSTs are more likely to be initiated and maintained in patients who are formally diagnosed with malnutrition, underscoring the utility of GLIM in identifying those most likely to benefit from NSTs. Additionally, 16.7% of non-malnourished participants developed malnutrition during the 90-day follow-up period, which may partly account for the higher rates of readmission and falls observed in this group. These findings indicate that even with adequate nutritional status at admission, older adults remain susceptible to nutritional deterioration due to factors such as intercurrent illness, appetite loss, or insufficient post-discharge support. This highlights the importance of ongoing nutritional monitoring and reinforces the need for routine re-assessment as a key element of standard geriatric care—even in those who are not initially identified as malnourished.

Previous studies have rarely focused on patients at nutritional risk who do not meet the GLIM criteria. Only one related study reported that NSTs reduced the rate of infectious complications in patients who were at nutritional risk (according to the NRS2002 score) but did not meet the GLIM criteria [25]. Unlike our study, above-mentioned study reevaluated a previously published prospective observational study, primarily involving surgical patients undergoing abdominal surgery, with parenteral nutrition being the predominant nutritional therapy. While nutritional screening tools have proven valuable for predicting clinical outcomes, they are yet to reliably determine the patients that would benefit the most from NSTs. This raises the question of whether malnutrition diagnosed by the GLIM criteria compared with nutritional risk identified using the NRS2002 score serves as a better indication for NSTs. We are concerned that NSTs may result in excessive nutritional intervention with a poor cost-effectiveness ratio in some patients, particularly older inpatients, among whom nutritional risk is more prevalent. Malnutrition in older patients is influenced by multiple underlying conditions, disease severity, and inflammatory responses, all of which impact the effectiveness of NSTs [26].

The ESPEN guideline on nutritional support for medical inpatients with multimorbidity emphasizes the need to address patient heterogeneity and consider acute phase responses (assessed by clinical presentation and hsCRP) along with underlying disease when initiating nutritional support [ [27]]. The GLIM criteria recognize that disease-related malnutrition can arise from either inflammation- or undernutrition-driven catabolism. A better understanding of the phenotype and etiology of malnutrition, reflecting specific pathophysiological mechanisms, may improve patient characterization and enable better selection of those most likely to benefit from NSTs, contributing to a more personalized approach. High-quality randomized trials are still needed to provide stronger evidence in the future.

The present study further supports the clinical applicability of the GLIM criteria in hospitalized older inpatients. Malnourished patients identified by the GLIM framework demonstrated significant improvements in both nutritional status and functional capacity—including BMI, MNA-SF score, calf circumference, and Barthel ADL index—following individualized NSTs. These improvements are considered clinically meaningful in the context of geriatric care. Importantly, the observed reductions in hospital readmission and fall incidence suggest that early nutritional intervention guided by GLIM may contribute to safer care transitions and delayed functional decline. From a clinical perspective, our findings highlight the need to incorporate GLIM-based nutritional assessment into routine geriatric evaluation. A standardized care pathway—including early screening, GLIM confirmation, personalized NSTs, and sustained follow-up—may help improve overall health outcomes in malnourished older patients. Further studies are warranted to evaluate the long-term impact of such interventions on clinical recovery, independence, and quality of life.

Our study has certain limitations. First, due to the involvement of multiple centers, coordinating the data for patients screened and excluded was difficult. Consequently, we could not determine the total number of screened patients. Second, the brief follow-up duration led to a low occurrence of key study outcomes, such as death, readmission, new infections, and falls. Thus, establishing a long-term follow-up program is essential to evaluate the effects of NSTs on clinical outcomes more thoroughly. Finally, the overlap of our follow-up period with the coronavirus disease 2019 pandemic in China prevented many older patients from attending hospital outpatient clinics due to associated restrictions, resulting in lost follow-up data.

## 5 Conclusions

Among hospitalized older inpatients at nutritional risk, those diagnosed with malnutrition according to the GLIM criteria showed significant improvements in both nutritional and functional statuses following NSTs, along with reduced risks of readmission and falls. These findings reinforce the predictive value of the GLIM-based diagnostic framework and advocate for its integration into routine clinical practice to better identify older inpatients most likely to benefit from targeted nutritional interventions.

## CRediT authorship contribution statement

Conceptualization: Xiaohong Liu, Shan Jiang, and Jiaojiao Li. Data curation: Shan Jiang and Xiaohong Liu. Formal analysis: Shan Jiang, Jiaojiao Li, and Xiaohong Liu. Investigation: Xiling Chen, Lan Ma, Qihao Guo, Yuehui Wang, Xuan Qu, Liping An, Lan Luo, Wei Huang, Yonghua Wu, Hongyu Zhang, Cuntai Zhang, Yun Fan, Songbai Zheng, and Jian Cao. Methodology: Xiaohong Liu, Shan Jiang, and Jiaojiao Li. Project administration: Xiaohong Liu and Shan Jiang. Supervision: Shan Jiang, Xiling Chen, Lan Ma, Qihao Guo, Yuehui Wang, Xuan Qu, and Liping An. Writing – original draft: Shan Jiang, Xiaohong Liu. Writing – review & editing: Shan Jiang, Xiling Chen, Lan Ma, Qihao Guo, Yuehui Wang, Xuan Qu, Jiaojiao Li, and Liping An.

## Funding

This work was supported by the Bethune Charitable Foundation [grant number X-J-2020-017]. The Bethune Charitable Foundation was not involved in the study design, data collection or preparation of the manuscript.

## Declaration of competing interest

The authors declare no conflicts of interest.
